# Oral microbial dysbiosis precedes development of pancreatic cancer

**DOI:** 10.1080/20002297.2017.1374148

**Published:** 2017-09-18

**Authors:** Ingar Olsen

**Affiliations:** ^a^ Department of Oral Biology, Faculty of Dentistry, University of Oslo, Oslo, Norway

In the United States, more than 50,000 people will acquire pancreatic cancer this year. This cancer type is the fourth leading cause of cancer deaths in the United States [1]. Less than 10% of those diagnosed will be alive in 5 years [2].

Recently, a prospective nested case-control study showed that carriage of *Porphyromonas gingivalis* and *Aggregatibacter actinomycetemcomitans*, two keystone pathogens in periodontitis, and a decreased relative abundance of the phylum Fusobacteria and its genus *Leptotrichia*, related to an increased risk of pancreatic cancer [3]. The study was based on oral mouth wash samples from 361 men and women with incident adenocarcinoma of pancreas and 371 matched healthy controls from two prospective cohort studies. The study was controlled for variances in age, race, sex, smoking status, alcohol use, body mass index, and diabetes. The microbiota of the oral wash samples was characterized with *16S rRNA* gene sequencing. The relationship between the total oral microbiota and pancreatic cancer was analyzed by weighted and unweighted UniFrac distances [4]. These tests evaluate the phylogenetic similarity of bacterial community pairs, taking into account relative abundance or presence/absence, respectively, of operational taxonomic units (OTUs). When the overall microbiota composition was compared initially, no differences in either cohort could be seen, with respect to overall phylogenetic distance of oral microbiome composition, between subjects who developed pancreatic cancer and those who did not. The participants were monitored for almost a decade to determine who developed pancreatic cancer. Participants with *P. gingivalis* in their microbiome had a 59% higher risk of developing cancer than those without [2]. Patients with *A. actinomycetemcomitans* had at least 50% increased risk of developing pancreatic cancer [2]. However, the association for *A. actinomycetemcomitans* was not as statistically strong as for *P. gingivalis*. The latter was also found in the oral microbiomes of control persons, but not as frequent as in pancreatic cancer patients (26% vs 35%). For comparison, 4% of control patients and 9% of pancreatic cancer patients carried *A. actinomycetemcomitans*. The risks identified for these two species could not be associated with other species. The phylum Fusobacteria and its genus *Leptotrichia* were related to a decreased risk of pancreatic cancer. What was particularly noticeable in this study was that oral microbial dysbiosis preceded the development of pancreatic cancer. Oral samples were used that had been collected up to 10 years before cancer diagnosis. The increased risk was the same even after excluding pancreatic cancer cases that occurred less than 2 years after the samples were collected. This made it unlikely that the oral dysbiosis occurred after or simultaneous with the cancer development. It indicated that the oral microbiota had an etiological role in pancreatic cancer.

Although this was the first study to count bacteria directly in oral samples collected before onset of the disease, several previous epidemiological studies have suggested an association between periodontal disease/tooth loss and increased risk of pancreatic cancer [5–9]. A large European cohort found high serum antibodies to *P. gingivalis* (>200 ng/mL), and these antibodies were associated with a twofold increased risk for pancreatic cancer compared to those with lower levels of such antibodies [10]. Furthermore, in a prospective cohort study of over 50,000 male health professionals who constituted a homogenous socioeconomic population, a history of periodontitis was associated with a 64% increased risk for developing pancreatic cancer. This association was stronger for men who had never smoked, although smoking is generally regarded as a risk factor for pancreatic cancer [7]. Interestingly, a dense multispecies bacterial biofilm including oral bacteria was detected within the pancreatic duct of patients with calcific pancreatitis by using fluorescence *in situ* hybridization [11]. Clearly, oral bacteria can reach pancreatic sites through blood dissemination and also by swallowing.

How *P. gingivalis* and *A. actinomycetemcomitans* can contribute to pancreatic cancer is not clear. As pointed out by Fan et al. [3], *P. gingivalis* has considerable potential to evade the immune system by invading host cells and by disrupting signaling pathways through cytokine and receptor degradation. *A. actinomycetemcomitans* can activate Toll-like receptor (TLR) signaling pathways, and TLR9 has protumorigenic effects on pancreatic carcinoma [12]. *P. gingivalis* and *Fusobacterium nucleatum* have been shown to have a strong oral carcinogenic potential *in vitro* and in animal studies [13,14]. They are thought to contribute to oral carcinogenesis through inhibition of apoptosis, activation of cell proliferation, promotion of cellular invasion, induction of chronic inflammation, and production of carcinogens [15].

Fan et al. [3] found it premature to conclude that the two periodontal pathogens cause pancreatic cancer, although there was a clear indication that they did so. They also suggested that pancreatic and oral conditions could be related to an independent systemic inflammatory process. If so, then the oral bacteria might just be markers for the susceptibility to inflammation.

Consequently, more studies are needed and will be performed to establish whether, and if so how, oral microbial dysbiosis causes pancreatic cancer. As a general recommendation, I feel that more efforts should be made now to prove if, and how oral microorganisms can cause the large number of systemic diseases they have been associated with..
